# Digital Interventions for Patients With Juvenile Idiopathic Arthritis: Systematic Review and Meta-Analysis

**DOI:** 10.2196/65826

**Published:** 2025-03-21

**Authors:** Zihan Ren, Yawen Chen, Yufeng Li, Panyu Fan, Zhao Liu, Biyu Shen

**Affiliations:** 1 School of Design, Shanghai Jiao Tong University, 800 Dongchuan Road, Minhang District, Shanghai, 200240, China, 86 18801971294; 2 Shanghai Children's Medical Center, School of Medicine, Shanghai Jiao Tong University, Shanghai, China

**Keywords:** juvenile idiopathic arthritis, digital intervention, application, children health care, pediatrics

## Abstract

**Background:**

Juvenile idiopathic arthritis (JIA) is a chronic rheumatic condition requiring long-term, multidisciplinary treatment, which consumes significant health care resources and family energy. This study aims to analyze the effectiveness of digital interventions on patient outcomes in individuals with JIA.

**Objective:**

This meta-analysis aimed to evaluate the impact of digital interventions on alleviating symptoms and improving overall well-being in children and adolescents with JIA.

**Methods:**

A systematic search of 5 databases identified randomized controlled trials assessing the impact of digital interventions on physiological and psychological outcomes in adolescents and children (average age ≤19 y). Outcomes included pain, physical activity, health-related quality of life, self-efficacy, and disease-related issues. A total of 2 reviewers independently screened papers and extracted data on intervention functionalities and outcomes, assessing the risk of bias. A meta-analysis using a random-effects model synthesized the results.

**Results:**

The review included 11 studies involving 885 patients with JIA. Digital interventions included educational (eg, self-management training), therapeutic (eg, pain management), and behavioral (eg, promoting physical activity) approaches. These were delivered through websites, telephone consultations, video conferences, apps, and interactive games, with durations ranging from 8 to 24 weeks and no clear link observed between intervention length and outcomes. Compared with conventional control groups, digital interventions were significantly effective in alleviating pain (standardized mean difference [SMD] −0.19, 95% CI −0.35 to −0.04) and enhancing physical activity levels (SMD 0.37, 95% CI 0.06-0.69). Marginal improvements in health-related quality of life, self-efficacy, and disease-related issues were observed, but these did not reach statistical significance (SMD −0.04, 95% CI −0.19 to 0.11; SMD 0.05, 95% CI −0.11 to 0.20; and SMD 0.09, 95% CI −0.11 to 0.29, respectively). The Grading of Recommendations, Assessment, Development, and Evaluation (GRADE) approach rated the quality of evidence for pain, health-related quality of life, self-efficacy, and disease-related issues as moderate, while the evidence quality for physical activity was assessed as low.

**Conclusions:**

Digital interventions can alleviate pain and enhance physical activity in patients with JIA. However, given the limited sample size and high risk of bias in some studies, further high-quality research is needed to improve the treatment and management of JIA.

## Introduction

Juvenile idiopathic arthritis (JIA) is a prevalent chronic rheumatic ailment affecting children, causing joint pain and inflammation that can disrupt their daily lives [1]. During flare-ups, it can hinder academic performance, social interactions, and normal activities [2], while the complexity of treatment and associated complications further strain health care systems and drive up costs [3-6]. Since JIA requires ongoing monitoring and treatment [7,8], patients face a lifelong responsibility for managing the disease as they grow older [9]. Consequently, patients are encouraged to actively engage in lifestyle modifications and health-related decision-making [10]. Physical activity, including aerobic fitness and strength training, is recognized as a helpful nondrug intervention, offering potential benefits in improving overall well-being and lessening the impact of JIA symptoms [11-13].

Internet-based digital tools, including mobile applications, websites, and other platforms, have become essential components of nonpharmaceutical interventions. These tools enable remote interaction and offer timely responses, making health care resources more accessible [14]. They can provide tailored rehabilitation interventions for pediatric chronic diseases and transitional care [15], such as fostering healthy behavioral habits through social media–based peer coaching [16]. Several mobile medical applications have been developed for adolescents and young individuals with JIA [17-22]. However, the research on their effectiveness has yielded varied results. While some studies have shown promising outcomes in terms of pain alleviation and physical function improvement, others have not replicated these results [22-24]. This variability in research findings highlights the need for further investigation and systematic evaluation to better understand the most effective components and digital health solutions in this domain, ensuring an accurate assessment of the evidence.

To date, previous reviews have assessed the effectiveness of mobile and e-medical interventions in aiding children and adolescents with JIA [25,26]. However, existing reviews have not focused on analyzing randomized controlled trials (RCTs), which could yield more precise results and reduce heterogeneity. The inclusion of only a minimal number of relevant outcomes (n≤3) in some meta-analyses, such as physical activity, limits the interpretation of findings cautiously and results in the absence of an effective theory of digital interventions for patients with JIA. Consequently, it remains unclear whether such interventions enhance clinical outcomes. Furthermore, as research on mobile medical interventions for JIA patients continues to evolve, it is essential to promptly integrate new research findings. This underscores the necessity for a fresh comprehensive evaluation of clinical interventions in this domain.

Therefore, our study aims to address these gaps by conducting a thorough analysis of digital interventions and their impact on clinical outcomes for patients with JIA.

## Methods

### Overview

This study follows the guidelines published in Preferred Reporting Items for Systematic reviews and Meta-Analyses [27] and the Cochrane Handbook of Systematic Reviews [28]. The priori protocol for the review is published in the International Prospective Register of Systematic Reviews (PROSPERO CRD42023471223).

### Search Strategy

The research was conducted with the guidance and support of institutional librarians. A subject-specific librarian, along with researchers ZR and YC, developed a search strategy without language restrictions, which was used to conduct a comprehensive search in PubMed, Embase, Cochrane Library, Ovid, and Medline [29], covering records from the earliest available to the latest date. The search used Boolean operators in combination with Medical Subject Headings terms and free-text keywords to identify studies on the impact of digital interventions on JIA. The following search string was used as an example: (“Juvenile Idiopathic Arthritis” OR “Pediatric Rheumatic Diseases” OR “Juvenile Chronic Arthritis”) AND (“mHealth” OR “Digital Health” OR “Mobile Health”) AND (“Randomized Controlled Trial” OR “RCT” OR “Clinical Trial”). The full search strategy is provided in Multimedia Appendix 1, the specific keywords used for the search are provided in Multimedia Appendix 2.

The studies identified through this strategy were managed through the literature management software, Zotero (Corporation for Digital Scholarship). The 2 authors, ZR and YC, screened the identified studies, in line with predefined inclusion and exclusion criteria. Any discrepancies during this process were resolved through discussion between the researchers. Multimedia Appendix 1 shows the detailed search formulas. RCTs of any design, including crossover, multicenter, and cross-over trials, that were published in English are included in this review.

### Participants

Episodes of JIA typically manifest in individuals before the age of 16 years [30]. However, considering the chronic nature of the condition and the need for ongoing treatment, the minimum age for inclusion in international pediatric treatment reference populations has risen to an average of 18.7 (SD 2.6) years.

Hence, for the purpose of this review, the term “children and adolescents” refers to individuals between the ages of 1 and 19 years [31]. The study population comprises children and adolescents diagnosed with JIA by a rheumatologist, ranging from 1 to 19 years old. Infants and neonates under the age of 1 year were excluded from the study population.

### Intervention

In assessing the effectiveness of interventions for JIA recovery, the study focused on digital platforms such as somatic gaming, smart applications, teleconferencing, televideo, and health websites.

### Control Condition

All types of control groups were considered in this study, including waitlists, physical therapy or minimal intervention groups, alternative treatments, and standard care delivered through web-based health care websites and apps. For example, the control group may use platforms like jong-en-reuma.nl, which provides information on medical issues and emotional support [32].

### Outcome

#### Primary Outcome

There were 2 primary outcomes: pain (47-item Recalled Pain Inventory and 11-point Numeric Rating Scale) and physical activity (7-day activity diary [subjective], accelerometer [objective measurement], and Duruoz Hand Index Questionnaire.

#### Secondary Outcome

There were 2 secondar outcomes: health-related quality of life (Juvenile Arthritis Quality of Life Questionnaire, Pediatric Quality of Life Arthritis Module, Dutch Consensus Health Assessment Questionnaire Disability Index, and Pediatric Quality of Life Inventory [version 4.0]), self-efficacy (Children’s Arthritis Self-Efficacy scale and Dutch Arthritis Self-Efficacy Scale), and illness-related issues (Medical Issues, Exercise, Pain, and Social Support questionnaire).

### Risk of Bias

Risk of bias was assessed using the risk of bias tool of the Cochrane Handbook for Systematic Reviews [28]. Quality of evidence for outcomes was assessed according to the 5 Grading of Recommendations, Assessment, Development, and Evaluation (GRADE) domains, including study limitation (risk of bias), inconsistency, indirectness, imprecision, and publication bias [33]. The bias was assessed by the 2 independent authors, ZR and YL. Any discrepancies were resolved through discussion and reexamination within the research group.

### Data Extraction and Analysis

In order to identify and present common statistical descriptions of methodological heterogeneity, a descriptive integrated methodology was used. All findings were interpreted within the context of each study, considering the total number of studies and the assessed risk of bias. Using Review Manager (RevMan) software (version 5.4; Cochrane), this study conducted a random-effect meta-analysis to compare the standardized mean difference (SMD) for parameters across at least 3 studies between patients receiving general care and those using internet-based interventions. SMD and 95% CI were calculated using baseline and study end scores inputted into RevMan 5.4. Forest plots were generated using random-effect models for continuous data, presenting a summary of the effect distribution. Cohen’s general rule of experience was applied, where an SMD of 0.2 signifies a “small” effect, 0.5 denotes a “moderate” effect, and 0.8 indicates a “large” effect. Furthermore, subgroup analysis was conducted to assess the impact of professional caregivers and intervention tools on the efficacy of e-medical intervention outcomes. Heterogeneity within the compiled studies was evaluated using *I*^2^ statistics, and the carat test was used to assess significance. Heterogeneity levels were classified as low (*I*^2^<25%; *P*>.1), moderate (*I*^2^=25%‐49%), or high (*I*^2^>50%).

## Results

### Literature Selection

**Figure 1.**
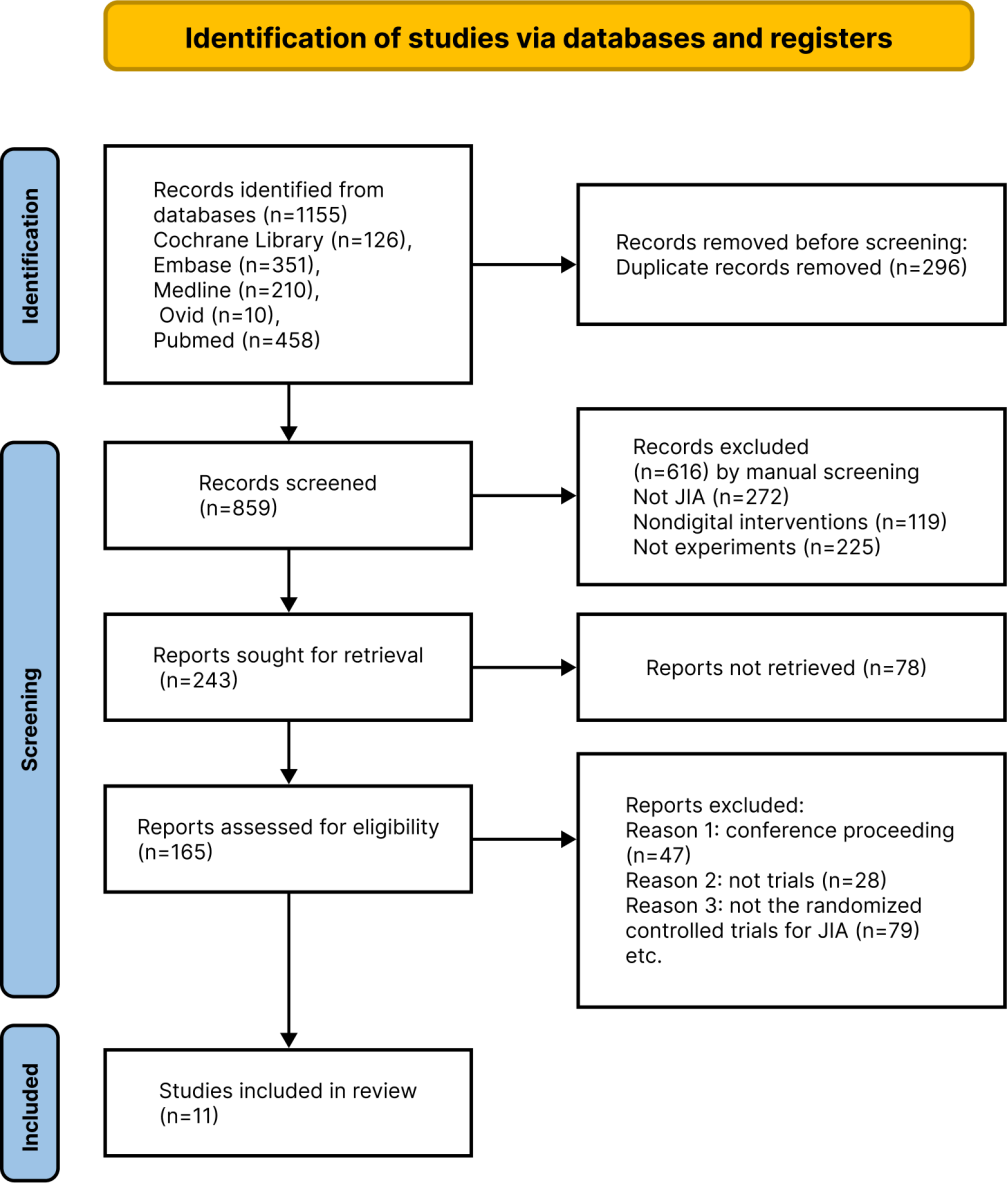
Summary of the study selection process using the PRISMA (Preferred Reporting Items for Systematic Reviews and Meta-Analyses). JIA: juvenile idiopathic arthritis.

We initially identified 1155 studies. After excluding duplicate studies (n=296) and irrelevant studies (n=694), 165 studies remained for abstract evaluation. A total of 154 studies were excluded for the following reasons: conference proceedings (n=47), not trials (n=28), and not RCTs for JIA (n=79). Ultimately, 11 RCTs were included in the meta-analysis. The screening process is illustrated in Figure 1.

### Participant Statistics

Table 1 shows the population characteristics, interventions, outcomes and study types of the 11 studies. Of these studies, 3 were from the Netherlands [32,34,35], 5 from Canada [36-40], and 1 each in the United States, Switzerland, and Turkey [38,40,41]. These studies included 289 individuals, predominantly female children and adolescents (663/885, 74.9%). A variety of juvenile arthritis subtypes were observed, with oligoarthritis being the most prevalent subtype (259/885, 29.3%). Almost all studies, with the exception of one [42], accounted for disease activity. The duration of the disease since diagnosis was documented in the majority of studies (7/11) [32,35-37,39,40,42].

**Table 1.** Population characteristics, interventions, outcomes, and study types of the 11 studies.

Author, country	Particular year	Average age (years)		Percentage of women, n/N (%)		Subtypes of JIA^c^	Participants		Outcomes	Type of study
		IG^a^	CG^b^	IG	CG		IG	CG		
Lelieveld et al [34], the Netherlands	2010	10.6 (1.5)^d^	10.8 (1.4)^d^	15/16 (88)	14/16 (88)	55% Persistent oligoarticular, 6% extended oligoarticular, 27% polyarticular, and 12% systemic	Internet-based meeting	—^e^	Physical activity level, number of days with ≥1 hour of moderate to intense activity per day, aerobic capacity, maximum heart rate, and resting heart rate.	Pilot randomized controlled trial
Stinson et al [36], Canada	2010	14.4 (1.3)^d^	14.8 (1.7)^d^	15/22 (68)	16/24 (67)	22% oligoarticular, 9% oligoarticular-extended, 24% polyarticular (RF–)^f^, 7% polyarticular (RF+), 7% systemic, 7% psoriatic, 20% enthesitis-related, and 7% Unknown or other	Internet intervention	Attention control	Healthy life-related quality (primary outcome), pain intensity, stress, knowledge, adherence, and self-efficacy (secondary outcomes)	Experimental randomized controlled trial
Stinson et al [37], Canada	2016	14.11 (1.53)^d^	14.42 (2.04)^d^	17/18 (94)	14/14 (100)	19% Polyarthritis (RF positive), 19% polyarthritis (RF negative), 3% polyarthritis (RF status unknown), 31% oligoarthritis, 25% psoriatic arthritis, and 3% enthesitis-related arthritis	Skype group	Standard care only	Feasibility (primary outcome) Self-management, self-efficacy, pain, social support, and health-related quality of life (secondary outcome)	Experimental randomized controlled trial
Ammerlaan et al [32], the Netherlands	2017	19.1 (2.7)^d^	19.1 (2.9)^d^	29/35 (83)	30/32 (94)	21% Oligo-articular JIA, 36% poly-articular JIA, 12% systemic JIA, and 31% other	Specific internet project intervention and designated website	Standard care and designated website	Self-efficacy (primary outcome), self-management, disease activity, health-related quality of life absence from courses , medication use, and adherence (secondary outcomes)	Randomized controlled trial
Armbrust et al [35], the Netherlands	2017	9.7 (8.7‐11.3)^g^	10.2 (9.0‐10.8)^g^	21/28 (75)	12/21 (57)	24% Persistent oligoarticular JIA, 14% extended oligoarticular JIA, 37% polyarticular JIA, 4% rheumatoid factor positive, 4% enthesitis-related JIA, 4% psoriasis-related JIA, and 12% systemic JIA	Internet intervention, school and physical education	Access to standard care, school and physical education	Physical activity (primary outcome), exercise capacity, healthy life–related quality, disease activity, functional capacity, pain and well-being, and school engagement (secondary outcomes)	Multicenter randomized controlled trial
Ramelet et al [43], Switzerland	2017	—^h^	—	8/14 (57)	6/10 (60)	29% enthesitis-related JIA, 5% undifferenciated JIA, 27% oligoarticular JIA, 7% polyarticular JIA, 2% systemic JIA, and 30% other	Medical and telephone care consultations	Medical consultations only	Satisfaction (primary outcome), morning stiffness, and pain (secondary outcome)	Cross-over randomized clinical trial
Arman et al [42], Turkey	2019	12.36 (2.98)^d^	13.16 (3.35)^d^	21/25 (84)	21/25 (84)	44% oligoarticular JIA and 56% polyarticular JIA	Practice everyday activities with video-based games (Xbox 360 Kinect)	Practice daily activities with real-life materials	Upper extremity function (primary outcome), pain, upper extremity muscle strength, grip and pinch strength, and time-based activity performance (secondary outcome)	Randomized clinical trial
Chadi et al [38], Canada	2019	15.4 (13-18)^i^	15.2 (13-17)^i^	7/9 (78)	7/9 (78)	—	Take video conferencing courses at home	Take offline courses in hospital	Acquisition of positive thinking skills (primary outcome), mood and anxiety, self-esteem, illness perception, salivary cortisol changes (secondary outcome)	Pilot randomized controlled trial
Connelly et al [41], United States	2019	14.6 (1.8)^d^	14.5 (1.7)^d^	98/144 (68)	111/145 (77)	21% Oligoarticular (extended or persistent), 45% polyarticular (RF-, RF+ ,or RF unknown), 34% other (enthesitis-related JIA, psoriatic, systemic, and undifferentiated)	Teens taking charge	An educational website	Pain interference and intensity, health-related quality of life (primary outcome), self-efficacy, pain coping, emotional regulation, and condition knowledge (secondary outcome)	Multicenter randomized clinical trial
Stinson et al [39], Canada	2020	14 (1.5)^d^	14.5 (1.7)^d^	63/88 (72)	91/131 (70)	2% Systemic, 21% oligoarthritis, 11% oligoarthritis—extended, 23% polyarthritis (RF-), 9% polyarthritis (RF+), 11% psoriatic arthritis, 16% enthesitis-related arthritis, 4% undifferentiated, and 3% other	Specific website and phone consultations	Public website and telephone consultation	Pain intensity, pain interference and HRQL^j^ (primary outcomes), emotional symptoms, compliance, coping, knowledge, and self-efficacy (secondary outcomes)	Randomized controlled trial
Lalloo et al [40], Canada	2021	14.9 (1.7)^d^	15.1 (1.6)^d^	23/29 (79)	24/31 (77)	5% Systemic, 16% oligoarthritis, 9% oligoarthritis-extended, 24% polyarthritis (RF–), 5% polyarthritis (RF+), 12% psoriatic arthritis, 21% enthesitis-related arthritis, 5% undifferentiated, and 3% other	iCanCope application version, including symptoms tracking and other functions	A version of icancope that only contains the symptom following feature	Participant accrual and attrition rates, success rate of app deployment, acceptability and compliance (primary outcomes), pain intensity, pain-related activity limitations, and health-related quality of life (secondary outcomes)	Randomized controlled trial

a IG: intervention group.

b CG: control group.

c JIA: juvenile idiopathic arthritis.

d Mean (SD).

e Not available.

f RF: rheumatoid factor.

g Median (IQR).

h This study only showed the mean age of the overall group (13.1 years).

i Median (age range).

j HRQL: health-related quality of life.

### Intervention Group

#### Overview

Table 2 demonstrates main digital tools and methods. A total of 6 studies implemented internet-based physical activity intervention programs, health management websites, and telephone support initiatives. Among these, 4 studies included routine telephone consultations and interviews. In addition, 1 study used video conferences for skills training [38], while another used self-management pain applications on mobile phones for experimentation [40]. In addition, an emotional games-based task-oriented activity training study was conducted [42]. All interventions examined lasted at least 8 weeks, with the longest intervention cycle spanning 18 months [35].

**Table 2.** Main intervention methods.

Author, country	Digital tools or methods					Duration of intervention
	Specific websites	Telephones	Videoconferencing	Application	Somatosensory game	
Lelieveld et al [34], the Netherlands	✓					17 weeks
Stinson et al [36], Canada		✓				12 weeks
Stinson et al [37], Canada		✓				8 weeks
Ammerlaan et al [32], the Netherlands	✓					24 weeks
Armbrust et al [35], the Netherlands	✓					18 months
Ramelet et al [43], Switzerland		✓				12 months each
Arman et al [42], Turkey					✓	8 weeks
Chadi et al [38], Canada	✓		✓			8 weeks
Connelly et al [41], United States	✓					12 weeks
Stinson et al [39], Canada	✓	✓				12 weeks
Lalloo et al [40], Canada				✓		8 weeks

#### Functional Classification of Interventions

Digital interventions for patients with JIA are versatile, serving multiple functions. The purposes of these interventions include promoting physical activity (n=4), facilitating self-management for establishing healthy habits and reaching milestones (n=4), providing education on disease and health-related knowledge (n=8), offering stress relief to improve mood (n=4), and enhancing communication skills for better integration into school and society (n=7). Furthermore, half of the studies (n=7) supplemented the digital intervention program with telephone and video communication to augment its positive impact on children.

#### Statistics of Digital Interventions

A total of 8 studies used internet-based interventions based on previously developed projects or applications (Table 3). In addition, 3 studies used the Teens Taking Charge website as an intervention [36,39,41]. Furthermore, 2 studies used Rheumates@Work as an intervention [34,35]. For the experimental group’s digitization project, 1 study used iPeer2Peer [37], Challenge your arthritis [32], and iCanCope [40]. Of all the intervention schemes, only 1 study referenced the theoretical framework as nursing guidance for their intervention schemes [43]. Care assessments conducted by nurses were guided and documented using Cox’s interaction model of client health behavior [44] to ensure the continuity of care for children and their families.

**Table 3.** Names and functions of digital intervention tools.

Author, country	Intervention project name	Main functions					Additional functions
		Promote physical activity	Set goals	Health education	Manage emotions	Integrate into school or society	Video or phone consultation
Lelieveld et al [34], the Netherlands	Rheumates@Work	✓		✓			
Stinson et al [36], Canada	Teens Taking Charge	✓	✓	✓	✓	✓	✓
Stinson et al [37], Canada	iPeer2Peer			✓	✓	✓	✓
Ammerlaan et al [32], the Netherlands	Challenge your arthritis		✓			✓	
Armbrust et al [35], the Netherlands	Rheumates@Work	✓		✓		✓	
Ramelet et al [43], Switzerland	—^a^			✓		✓	✓
Arman et al [42], Turkey	Xbox 360 Kinect	✓					
Chadi et al [38], Canada	—			✓	✓		✓
Connelly et al [41], United States	Teens Taking Charge			✓	✓		✓
Stinson et al [39], Canada	Teens Taking Charge		✓			✓	✓
Lalloo et al [40], Canada	iCanCope		✓	✓		✓	

a Not available.

While enhancing self-management skills is vital for facilitating health care transition [45], only 2 RCTs [32,37] explicitly reported on self-management outcomes. The remaining articles primarily integrated self-management as a core component of digital interventions, with considerations on health education, goal setting, and mood management.

### Control Group

One study in this review did not specify the care received by the control group [34]. A total of 5 studies compared the intervention group to a control group that received standard or offline care (without internet and eHealth interventions) [35,37,38,42,43]. One study solely used telephone coaching communication [36]. A total of 4 studies compared a control group using a public website or eHealth with limited functionality to an intervention group receiving a specific digital design program [32,39-41]. The control groups in all trials assessed patients’ results at pretest and posttest.

### Risk of Bias Assessment

**Figure 2.**
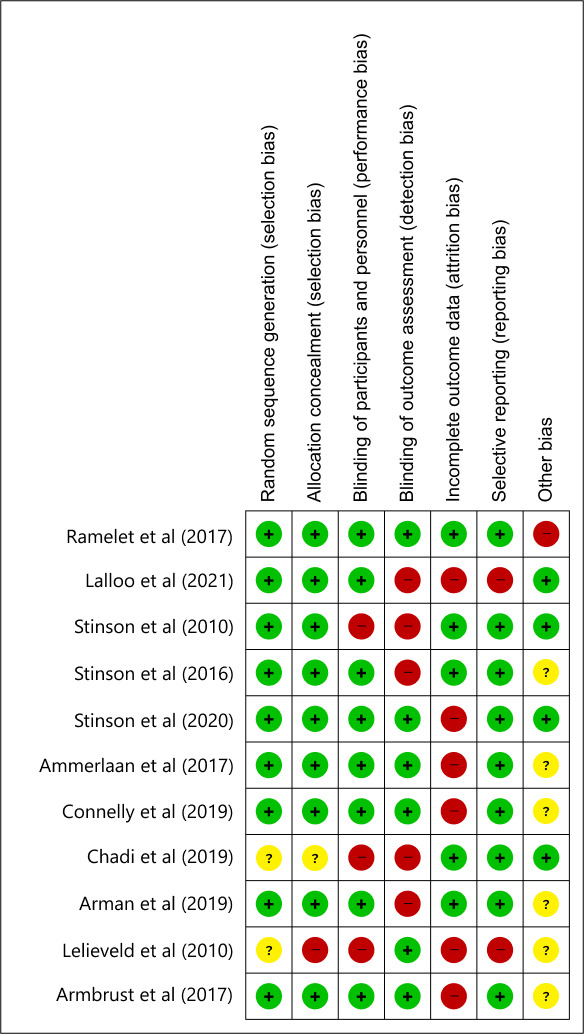
Risk of bias summary [32,34-43].

The results of the risk of bias assessment indicate that the criteria most commonly unmet were the blinding of outcome assessment and the adequacy of outcome data (Figure 2). Half of the studies (5/11 and 6/11) were deemed to have a high risk of bias in these 2 categories. In contrast, studies concerning randomized sequence generation were predominantly evaluated as having a low risk (9/11). Furthermore, 7 studies exhibited a medium risk of bias, while 4 studies were categorized as having a high risk of bias.

### Quality of Evidence Rating

Table 4 presents the key comparative results with GRADE ratings. A total of 3 primary outcomes are rated as moderate quality, while 2 primary outcomes are rated as low quality.

**Table 4.** Main comparative findings and recommendation grading.

Author (Year of publication)	Outcome	OR^a^ (95% CI)	Studies (patients), n	Risk of bias	Inconsistency	Indirectness	Inaccuracy	Publication bias	Quality of evidence
Stinson et al [36] (2010), Stinson et al [37] (2016), Ammerlaan et al [32] (2017), Connelly et al [41] (2019), and Stinson et al [39] (2020).	Pain	−0.19 (−0.35 to −0.04)	5 (653)	Downgrade (High risk of incomplete data)	Nondegradation	Nondegradation	Nondegradation	Nondegradation	Moderate
Lelieveld et al [34] (2010), Ammerlaan et al [32] (2017), Armbrust et al [35] (2017), and Arman et al [42] (2019).	Physical activity	0.37 (0.06-0.69)	4 (160)	Downgrade (High risk of selective reporting)	One level down (low overlap)	Nondegradation	Nondegradation	Nondegradation	Low
Stinson et al [36] (2010), Stinson et al [37] (2016), Ammerlaan et al [32] (2017), Armbrust et al [35] (2017), Connelly et al [41] (2019), and Stinson et al [39] (2020).	Health-related quality of life	−0.02 (−0.17 to 0.13)	6 (702)	Downgrade (High risk of incomplete data)	Nondegradation	Nondegradation	Nondegradation	Nondegradation	Moderate
Stinson et al [36] (2010), Stinson et al [37] (2016), Ammerlaan et al [32] (2017), Connelly et al [41] (2019), and Stinson et al [39] (2020).	Self-efficacy	0.05 (−0.11 to 0.20)	5 (653)	Downgrade (High risk of incomplete data)	Nondegradation	Nondegradation	Nondegradation	Nondegradation	Moderate
Stinson et al [36] (2010), Stinson et al [37] (2016), Chadi et al [38] (2019), Connelly et al [41] (2019), and Stinson et al [39] (2020).	Disease-related issues	0.09 (−0.11 to 0.29)	5 (604)	Downgraded one level (high risk of blinding of outcome assessments)	One level down (low overlap)	Nondegradation	Nondegradation	Nondegradation	Low

a OR: odds ratio.

### Meta-Analysis Results

#### Pain

**Figure 3.**
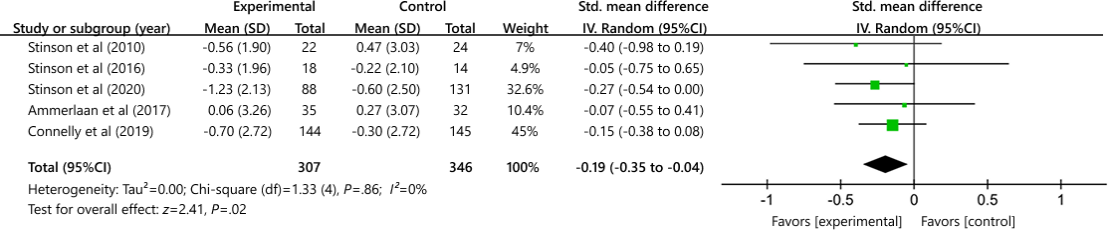
Effectiveness of digital health on pain outcomes [32,36,37,39,41].

Figure 3 depicts the impact of digital medical intervention on pain outcomes relative to all other control conditions. This analysis is based on findings from 5 studies involving 653 participants. A significant effect in favor of the intervention was observed (SMD −0.19, 95% CI −0.35 to −0.04; *P*=.86; *I*^2^=0%). Several studies posed a high risk of bias, resulting in a moderate GRADE rating for the quality of evidence after the intervention.

#### Physical Activity

**Figure 4.**
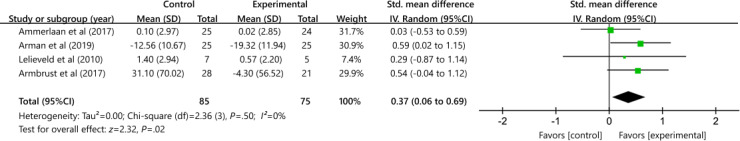
Effectiveness of digital health on physical activity outcomes [32,34,35,42].

Figure 4 demonstrates the results of the effectiveness of using digital health care on patients’ physical activity compared to using usual care and public websites. This analysis is based on findings from 4 studies involving 160 participants. The digital intervention had a statistically significant positive effect (SMD 0.37, 95% CI 0.06-0.69), and the results were not highly heterogeneous (*P*=.50; *I*²=0%). Several studies posed a moderate-to-high risk of bias and inconsistency, resulting in a low GRADE rating for the quality of evidence after the intervention.

#### Health-Related Quality of Life

**Figure 5.**
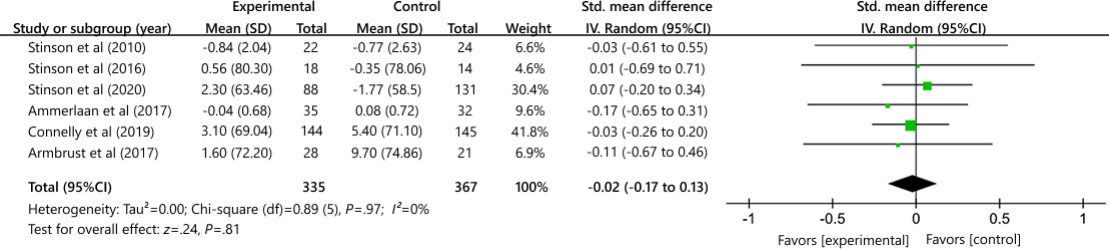
Effectiveness of digital health on health-related quality of life [32,35-37,39,41].

A total of 6 studies with 702 participants comparing digital interventions and control conditions did not show a difference in health-related quality of life between the 2 intervention conditions (SMD 0.02, 95% CI −0.17 to 0.13); heterogeneity (*P*=.97; *I*²=0%; Figure 5). Using the GRADE approach, the quality of evidence was rated moderate because of the high risk of bias in most studies (ie, incomplete data).

#### Self-Efficacy

**Figure 6.**
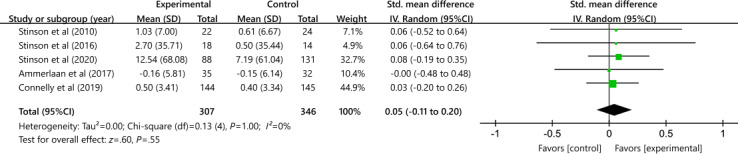
Effectiveness of digital health on self-efficacy outcomes [32,36,37,39,41].

A total of 5 studies with 653 participants comparing digital interventions and control conditions did not show a difference in self-efficacy between the 2 intervention conditions (SMD 0.05, 95% CI −0.11 to 0.20); heterogeneity (*P*=1.00; *I*²=0%; Figure 6). Using the GRADE approach, the quality of evidence was rated moderate because of the high risk of bias in most studies (ie, incomplete data).

#### Disease-Related Issues

**Figure 7.**
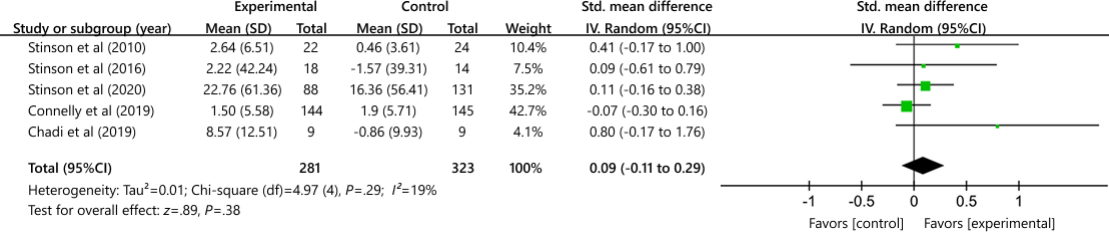
Effectiveness of digital health on disease-related issues [36-39,41].

Figure 7 demonstrates the effectiveness of interventions using digital health technology on patient outcomes for disease-related problems compared with other control conditions. A total of 5 studies included 604 participants (SMD 0.09, 95% CI −0.11 to 0.29) suggests that the effect is ultimately insignificant. The results exhibit minimal heterogeneity (*P*=.29, *I*²=19%). The evidence following the intervention was assessed as moderate in quality using the GRADE methodology, owing to the presence of bias risk and inconsistency across certain studies.

#### Subgroup Analysis

##### Effects of Peer Mentoring on Pain Outcome

**Figure 8.**
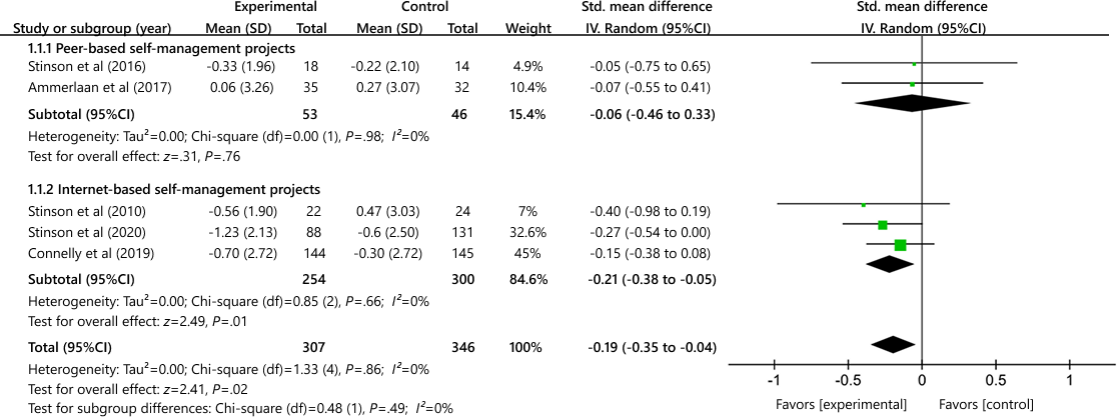
Effects of peer mentoring on pain outcomes [32,36,37,39,41].

The subgroup analysis revealed that the internet-based self-management program (n=3) resulted in a moderate effect size in pain reduction (SMD −0.21, 95% CI −0.38 to −0.05; heterogeneity *χ*²_2_=0.85; *P*=.66; *I*²=0%; Figure 8). However, our findings showed no significant effect of iPeer2Peer and Challenge Your Arthritis (n=2) on pain (SMD −0.06, 95% CI −0.46 to 0.33; heterogeneity *χ*²_1_=0.00; *P*=.98; *I*²=0%). Subgroup differences in pain outcomes were not significant between peer mentoring programs and other internet programs (*P*=.49; *I*²=0%).

##### Effects of Physicians on Disease-Related Issues Outcome

**Figure 9.**
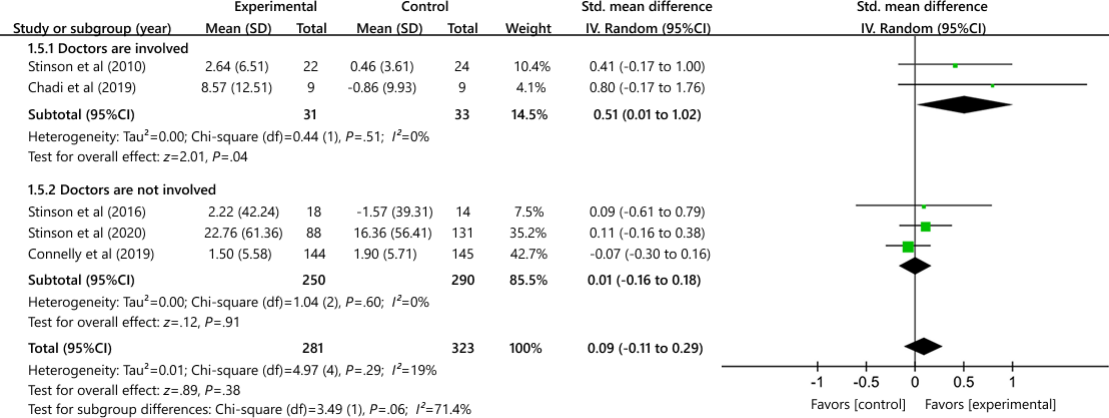
Effectiveness of e-health on disease-related issues when physicians are involved [36-39,41].

The disease-related issues in studies with physicians improve more than those without physicians as the main component of the intervention (SMD 0.51, 95% CI 0.01 to 1.02 and SMD 0.01, 95% CI −0.16 to 0.18, respectively; Figure 9). However, the difference was not statistically significant (SMD 0.09, 95% CI −0.01 to 0.29; heterogeneity *χ*²_4_=4.97; *P*=.29; *I*²=19%).

## Discussion

### Principal Findings

This systematic review comprehensively assessed studies on the effectiveness of digital interventions in aiding children and adolescents with JIA from physical and psychologically perspectives. According to the findings, patients who received digital medical technology interventions had significantly better physical activity outcomes (SMD 0.37, 95% CI 0.06-0.69) and experienced a reduction in pain outcomes (SMD −0.19, 95% CI −0.35 to −0.04) in comparison with those who received standard care. However, our research did not identify significant enhancements in disease-related issues (SMD 0.09, 95% CI −0.11 to 0.29), health-related quality of life (SMD −0.02, 95% CI −0.17 to 0.13), or self-efficacy (SMD 0.05, 95% CI −0.11 to 0.20).

### Primary Findings

#### Overview

The use of digital interventions delivered through the internet or mobile devices has expanded mental health practices for children and adolescents facing JIA in local contexts [46,47]. These interventions offer flexible training schedules, overcome constraints of space and time [48], ensure anonymity, and allow for behavioral adaptation. Nevertheless, our findings indicate that interventions using digital medical technology have a more pronounced impact on physiological outcomes, aligning with earlier research conducted by Butler et al [26]. This emphasis on physiological outcomes may be attributed to the inclusion of components targeting physical activity and motor skills in the interventions, such as fitness regimens, varied exercises, and intensive training. However, the interpretation of psychological outcomes is more complex, influenced by various factors including personal psychological state, social environment, and cultural background. In addition, achieving and sustaining psychological transformations often requires an extended period. While these potential reasons have not been examined, our findings indeed illuminate the distinct physiological and psychological effects of digital medical interventions, offering a new perspective for understanding and evaluating their merits. Further investigation is needed to compare the impacts of digital medical interventions on physiological and psychological outcomes, and to identify strategies for optimizing intervention effectiveness in diverse contexts.

#### Pain

Our findings demonstrated a notable reduction in pain-related outcomes following the implementation of digital interventions. Two of these studies focused on young patients with JIA transitioning to adult care facilities, who demonstrated high self-efficacy and positive attitudes. In addition, 3 studies implemented a telephone-based therapeutic communication intervention. Subgroup analysis outcomes revealed that patients using an internet-based self-management program (Teens Taking Charge) [36,39,41] experienced a great reduction in pain symptoms compared with those using a peer-directed self-management program [32,37]. These findings align with a pilot feasibility study on peer coaching for adolescents with chronic pain [18], where the control group showed superior pain reduction status. This discrepancy may be attributed to the absence of explicit pain symptom sections in the self-management programs examined, which focused instead on social relationships and goal-setting. In contrast, the control group’s website included comprehensive content addressing pain understanding and management, alongside audio and video features. In addition, Dennis et al [49] demonstrated that trained peer mentors could provide informational, evaluative, and emotional support to individuals with similar conditions, albeit without explicitly addressing pain relief. Hence, there is a need for studies about the usability of digital tools for managing pain symptoms in future research. These tools should go beyond mere documentation of pain symptoms and incorporate functionalities aimed at alleviating functional limitations, providing medication and exercise guidance, and offering strategies for managing low mood. Such enhancements are essential for improving the quality of life for patients coping with pain [50].

#### Physical Activity

Engaging in physical activity is essential for managing arthritis in patients [51]. Consistent with previous research findings [26], 4 findings emphasized the positive impacts of the internet interventions on physical activity. The majority of these studies incorporate clinically recommended activity training, which increases physical activity levels and improves endurance among patients. Studies suggest that individuals with arthritis can prevent disability and complications by promoting healthy physical activity throughout their lives [52]. However, as demand for face-to-face health care interventions for supporting physical activity adoption and maintenance increases, resource constraints become more pronounced [53]. In a previous study, serious games were used in joint rehabilitation for patients with JIA [54-56]. The findings indicated that these interventions led to increased levels of physical activity among the patients. Our findings support this observation, as 1 of the 4 studies using video games for task-oriented activity training [42] showed improvements in patient outcomes. However, concerns have arisen regarding potential inaccuracies in the effectiveness of exercise diaries and activity monitoring accelerometers used by children. Therefore, there is a need for more accurate methods of data acquisition. We advocate for the development of additional digital tools that integrate health education and physical activity-focused content.

### Secondary Findings

The secondary outcomes such as self-efficacy, health-related quality of life, and perception of disease-related issues did not show statistical significance. The previous research shows similar results. Lancaster et al [57] and Newby et al [58] did not find positive impacts of digital interventions on self-efficacy and quality of life. This discrepancy may be attributed to the measurement of self-efficacy which may not be adequately tailored to the conceptual, linguistic, and objective needs of children [32]. However, it is anticipated that improvements in quality of life may require more time to manifest [59], and changes might not be evident during shorter intervention periods. The Medical Issues, Exercise, Pain, and Social Support questionnaire, encompassing inquiries regarding medical matters, physical activity, psychological well-being, and social support [60], may experience compromised efficacy if a patient is insensitive to one of its components, indicating a limited awareness of disease-related concerns.

### Other Findings

It is worth noting that not all psychological interventions are ineffective. The subgroup findings show that when physicians are involved in intervention implementation, children and adolescents show improved understanding of disease-related issues. Previous research shows that online health communities involving both patients and health care providers can improve mental health in chronic conditions by allowing patients to consult and interact with physicians [61,62]. Physicians provide essential health knowledge, emotional support, and guidelines for the use of medical supplies, which is crucial for improving the health status of individuals with chronic conditions [63]. To improve intervention outcomes, digital interventions should incorporate features for real-time interaction with healthcare providers, enabling physicians to offer clinical insights and socioemotional support, thereby strengthening the doctor-patient relationship and improving health outcomes.

Furthermore, video-based mindfulness interventions have shown benefits for populations with chronic illnesses and other conditions [64,65]. A study comparing the efficacy of online mindfulness interventions and in-person interventions in enhancing the mental well-being of patients with JIA observed a notable decrease in anxiety and depression [38]. This reduction may be attributed to adolescents experiencing greater ease and relaxation in the familiar setting of their homes [66]. Furthermore, Voerman et al [67] found that digital interventions incorporating cognitive behavioral therapy led to significant improvements in the psychological and social outcomes of patients. Specifically, relaxation exercises and cognitive behavioral therapy effectively reduced pain frequency in children and adolescents, alleviating depressive symptoms and functional disorders [68]. Future investigations should aim to integrate a theoretical framework that addresses the psychological dimensions of the condition, ensuring a more comprehensive approach to intervention design.

### Limitations

Half of the studies (5/11) used digital tools that have been developed for over a decade, they may thus fail to represent the latest advancements in communication technologies and platforms. However, our findings indeed show their continued relevance and effectiveness. Second, the results of this review demonstrate that, from a statistical perspective, digital interventions are effective for certain patient outcomes. However, considering factors such as individual differences and variability in clinical environments, their clinical significance remains to be further validated. Future research should provide stronger evidence from a clinical perspective. Furthermore, the included studies are predominantly conducted in North American and European nations. As such, the findings of this analysis may not be universally applicable and may only offer insights into the integration of digital interventions within this specific population.

### Conclusions

This systematic review analyzes self-reported outcomes in patients with JIA, including pain, physical activity, quality of life, self-efficacy, and disease-related issues. The findings from 11 RCTs demonstrate that digital interventions significantly alleviate pain and improve physical activity. These results highlight the potential of digital tools to enhance JIA management and patient outcomes, providing a strong case for their integration into clinical practice. Future studies should consider the inclusion of physicians in digital interventions to better understand their impact on outcomes.

## Supplementary material

10.2196/65826 Multimedia Appendix 1 Search strategy.

10.2196/65826 Multimedia Appendix 2 Medical Subject Headings (MeSH) terms and free-text keywords.

10.2196/65826 Checklist 1 PRISMA (Preferred Reporting Items for Systematic reviews and Meta-Analyses) 2020 checklist.

## Abbreviations

GRADE: Grading of Recommendations, Assessment, Development, and Evaluation
JIA: juvenile idiopathic arthritis
RCT: randomized controlled trial
RevMan: Review Manager
