# Supplementary material for: Digital Interventions for Patients With Juvenile Idiopathic Arthritis: Systematic Review and Meta-Analysis
Source: JMIR Pediatr Parent. 2025 Mar 21;8:e65826. doi: 10.2196/65826 (PMC11952675; doi:10.2196/65826)
Supplement: Multimedia Appendix 1 [file pediatrics-v8-e65826-s001.docx]

Supplementary Material 1

# Search Strategy

MEDLINE: 1950 to 2024

Search Date: Dec 06 2024

| **#** | **Search Query** | **Results** |
| --- | --- | --- |
| 1 | TS=((Juvenile Idiopathic Arthritis) OR (Juvenile Rheumatoid Arthritis) OR (Juvenile Chronic Arthritis) OR (Pediatric Rheumatic Diseases) OR (Systemic Juvenile Idiopathic Arthritis) OR (Oligoarticular Juvenile Idiopathic Arthritis) OR (Polyarticular Juvenile Idiopathic Arthritis) OR (Enthesitis-Related Arthritis) OR (Psoriatic Juvenile Idiopathic Arthritis)) | 15319 |
| 2 | TS=((Telemedicine) OR (eHealth) OR (mHealth) OR (Mobile Applications) OR (Smartphone) OR (Videoconferencing) OR (Digital Health) OR (Internet-Based Intervention) OR (Remote Consultation) OR (Health Promotion/methods) OR (Patient Education as Topic/methods) OR (Self-Management) OR (Online Systems) OR (Health Information Technology) OR (Behavioral Medicine/methods) OR (Telehealth) OR (Digital Therapeutics)) | 389318 |
| 3 | #2 AND #1 | 210 |

PubMed

Search Date: Dec 06 2024

| **#** | **Search Query** | **Results** |
| --- | --- | --- |
| 1 | ((((Juvenile Idiopathic Arthritis[MeSH Terms]) OR (Juvenile Rheumatoid Arthritis[MeSH Terms])) OR (Juvenile Chronic Arthritis[MeSH Terms])) OR (Pediatric Rheumatic Diseases[MeSH Terms])) OR (Systemic Juvenile Idiopathic Arthritis[MeSH Terms]) | 15869 |
| 2 | (((Oligoarticular Juvenile Idiopathic Arthritis) OR (Polyarticular Juvenile Idiopathic Arthritis)) OR (Enthesitis-Related Arthritis)) OR (Psoriatic Juvenile Idiopathic Arthritis) | 16998 |
| 3 | #1 OR #2 | 20404 |
| 4 | (((((((Telemedicine[MeSH Terms]) OR (Mobile Applications[MeSH Terms])) OR (Smartphone[MeSH Terms])) OR (Videoconferencing[MeSH Terms])) OR (Health Promotion/methods[MeSH Terms])) OR (Patient Education as Topic/methods[MeSH Terms])) OR (Self-Management[MeSH Terms])) OR (Behavioral Medicine/methods[MeSH Terms]) | 119093 |
| 5 | ((((((((eHealth) OR (mHealth)) OR (Digital Health)) OR (Internet-Based Intervention)) OR (Remote Consultation)) OR (Online Systems)) OR (Health Information Technology)) OR (Telehealth)) OR (Digital Therapeutics) | 1008510 |
| 6 | #4 OR #5 | 1066337 |
|  | #3 AND #6 | 458 |

Embase

Search Date: Dec 04 2024

| # | Search Query | Results |
| --- | --- | --- |
| #1 | 'juvenile idiopathic arthritis'/exp OR 'juvenile idiopathic arthritis' OR 'juvenile rheumatoid arthritis'/exp OR 'juvenile rheumatoid arthritis' OR 'juvenile chronic arthritis'/exp OR 'juvenile chronic arthritis' OR 'pediatric rheumatic diseases' OR 'systemic juvenile idiopathic arthritis'/exp OR 'systemic juvenile idiopathic arthritis' OR 'oligoarticular juvenile idiopathic arthritis'/exp OR 'oligoarticular juvenile idiopathic arthritis' OR 'polyarticular juvenile idiopathic arthritis'/exp OR 'polyarticular juvenile idiopathic arthritis' OR 'enthesitis-related arthritis'/exp OR 'enthesitis-related arthritis' OR 'psoriatic juvenile idiopathic arthritis'/exp OR 'psoriatic juvenile idiopathic arthritis' | 30948 |
| #2 | 'digital therapeutics'/exp OR 'digital therapeutics' OR 'telemedicine'/exp OR 'telemedicine' OR 'ehealth'/exp OR 'ehealth' OR 'mhealth'/exp OR 'mhealth' OR 'mobile applications'/exp OR 'mobile applications' OR 'smartphone'/exp OR 'smartphone' OR 'videoconferencing'/exp OR 'videoconferencing' OR 'digital health'/exp OR 'digital health' OR 'internet-based intervention'/exp OR 'internet-based intervention' OR 'remote consultation'/exp OR 'remote consultation' OR 'health promotion/methods' OR 'patient education as topic/methods' OR 'self-management'/exp OR 'self-management' OR 'online systems'/exp OR 'online systems' OR 'health information technology'/exp OR 'health information technology' OR 'behavioral medicine/methods' OR 'telehealth'/exp OR 'telehealth' | 371073 |
| #3 | 'randomized controlled trial'/exp OR 'randomized controlled trial' OR 'clinical trial'/exp OR 'clinical trial' OR 'controlled clinical trial'/exp OR 'controlled clinical trial' OR 'double-blind method'/exp OR 'double-blind method' OR 'single-blind method'/exp OR 'single-blind method' OR 'random allocation'/exp OR 'random allocation' OR 'placebos'/exp OR 'placebos' OR 'treatment outcome'/exp OR 'treatment outcome' OR 'prospective studies'/exp OR 'prospective studies' OR 'cohort studies'/exp OR 'cohort studies' OR 'comparative effectiveness research'/exp OR 'comparative effectiveness research' OR 'multicenter study'/exp OR 'multicenter study' OR 'cross-over studies'/exp OR 'cross-over studies' OR 'follow-up studies'/exp OR 'follow-up studies' OR 'trial'/exp OR 'trial' OR 'research design'/exp OR 'research design' OR 'clinical trial, phase i' OR 'clinical trial, phase ii' OR 'clinical trial, phase iii' OR 'clinical trial, phase iv' | 12286909 |
| #4 | 'rct' OR 'randomized trial' OR 'controlled trial'/exp OR 'controlled trial' OR 'placebo-controlled trial' OR 'multicenter trial'/exp OR 'multicenter trial' OR 'single-center trial' OR 'prospective trial' OR 'cross-over trial'/exp OR 'cross-over trial' OR 'intervention trial'/exp OR 'intervention trial' OR 'trial study' OR 'cohort trial' OR 'open-label trial'/exp OR 'open-label trial' OR 'blinded trial' OR 'phase i/ii/iii/iv trial' OR 'evidence-based trial' | 11506232 |
| #5 | #3 OR #4 | 19186768 |
| #6 | #1 AND #2 AND #5 | 351 |

Ovid

Search Date: Dec 06 2024

| # | Search Query | Results |
| --- | --- | --- |
| 1 | (Juvenile Idiopathic Arthritis or Juvenile Rheumatoid Arthritis or Juvenile Chronic Arthritis or Pediatric Rheumatic Diseases or Systemic Juvenile Idiopathic Arthritis or Oligoarticular Juvenile Idiopathic Arthritis or Polyarticular Juvenile Idiopathic Arthritis or Enthesitis-Related Arthritis or Psoriatic Juvenile Idiopathic Arthritis).mp. [mp=title, book title, abstract, original title, name of substance word, subject heading word, floating sub-heading word, keyword heading word, organism supplementary concept word, protocol supplementary concept word, rare disease supplementary concept word, unique identifier, synonyms, population supplementary concept word, anatomy supplementary concept word] | 15043 |
| 2 | (Telemedicine or eHealth or mHealth or Mobile Applications or Smartphone or Videoconferencing or Digital Health or Internet-Based Intervention or Remote Consultation or Health Promotion/methods or Patient Education as Topic/methods or Self-Management or Online Systems or Health Information Technology or Behavioral Medicine/methods or Telehealth or Digital Therapeutics).mp. [mp=title, book title, abstract, original title, name of substance word, subject heading word, floating sub-heading word, keyword heading word, organism supplementary concept word, protocol supplementary concept word, rare disease supplementary concept word, unique identifier, synonyms, population supplementary concept word, anatomy supplementary concept word] | 117493 |
| 3 | 1 and 2 | 65 |
| 4 | 3 and "Randomized Controlled Trial" | 9 |
| 5 | 3 and "Clinical Trial" | 1 |
| 6 | 4 or 5 | 10 |

Cochrane

Search Date: Dec 05 2024

| # | Search Query | Results |
| --- | --- | --- |
| #1 | ((Juvenile Idiopathic Arthritis) OR (Juvenile Rheumatoid Arthritis) OR (Juvenile Chronic Arthritis) OR (Pediatric Rheumatic Diseases) OR (Systemic Juvenile Idiopathic Arthritis) OR (Oligoarticular Juvenile Idiopathic Arthritis) OR (Polyarticular Juvenile Idiopathic Arthritis) OR (Enthesitis-Related Arthritis) OR (Psoriatic Juvenile Idiopathic Arthritis)):ti,ab,kw AND ((Telemedicine) OR (eHealth) OR (mHealth) OR (Mobile Applications) OR (Smartphone) OR (Videoconferencing) OR (Digital Health) OR (Internet Based Intervention) OR (Remote Consultation) OR (Health Promotion) OR (Patient Education as methods) OR (Self Management) OR (Online Systems) OR (Health Information Technology) OR (Behavioral methods) OR (Telehealth) OR (Digital Therapeutics)):ti,ab,kw AND ((Randomized Controlled Trial) OR (Clinical Trial) OR (Controlled Clinical Trial) OR (Double Blind Method) OR (Single Blind Method) OR (Random Allocation) OR (Placebos) OR (Treatment Outcome) OR (Prospective Studies) OR (Cohort Studies) OR (Comparative Effectiveness Research) OR (Multicenter Study) OR (Cross Over Studies) OR (Follow Up Studies) OR (Trial) OR (Research Design) OR (RCT) OR (Randomized trial) OR (Controlled trial) OR (Placebo controlled trial) OR (Multicenter trial) OR (Single center trial) OR (Prospective trial) OR (Cross over trial) OR (Intervention trial) OR (Trial study) OR (Cohort trial) OR (Open label trial) OR (Blinded trial) OR (Evidence based trial)):ti,ab,kw (Word variations have been searched) | 126 |
