# Supplementary material for: Digital Interventions for Patients With Juvenile Idiopathic Arthritis: Systematic Review and Meta-Analysis
Source: JMIR Pediatr Parent. 2025 Mar 21;8:e65826. doi: 10.2196/65826 (PMC11952675; doi:10.2196/65826)
Supplement: Multimedia Appendix 2 [file pediatrics-v8-e65826-s002.docx]

Supplementary Material 2

# Mesh terms and free-text keywords

## Participants

(Juvenile Idiopathic Arthritis) OR (Juvenile Rheumatoid Arthritis) OR (Juvenile Chronic Arthritis) OR (Pediatric Rheumatic Diseases) OR (Systemic Juvenile Idiopathic Arthritis) OR (Oligoarticular Juvenile Idiopathic Arthritis) OR (Polyarticular Juvenile Idiopathic Arthritis) OR (Enthesitis-Related Arthritis) OR (Psoriatic Juvenile Idiopathic Arthritis)

## Interventions

(Telemedicine) OR (eHealth) OR (mHealth) OR (Mobile Applications) OR (Smartphone) OR (Videoconferencing) OR (Digital Health) OR (Internet-Based Intervention) OR (Remote Consultation) OR (Health Promotion/methods) OR (Patient Education as Topic/methods) OR (Self-Management) OR (Online Systems) OR (Health Information Technology) OR (Behavioral Medicine/methods) OR (Telehealth) OR (Digital Therapeutics)

## Type of Study

(Randomized Controlled Trial) OR (Clinical Trial) OR (Controlled Clinical Trial) OR (Double-Blind Method) OR (Single-Blind Method) OR (Random Allocation) OR (Placebos) OR (Treatment Outcome) OR (Prospective Studies) OR (Cohort Studies) OR (Comparative Effectiveness Research) OR (Multicenter Study) OR (Cross-Over Studies) OR (Follow-Up Studies) OR (Trial) OR (Research Design) OR (Clinical Trial, Phase I) OR (Clinical Trial, Phase II) OR (Clinical Trial, Phase III) OR (Clinical Trial, Phase IV) OR (RCT) OR (Randomized trial) OR (Controlled trial) OR (Placebo-controlled trial) OR (Multicenter trial) OR (Single-center trial) OR (Prospective trial) OR (Cross-over trial) OR (Intervention trial) OR (Trial study) OR (Cohort trial) OR (Open-label trial) OR (Blinded trial) OR (Phase I/II/III/IV trial) OR (Evidence-based trial)
